# Factor structure of the Oxford Shoulder Score: secondary analyses of the UK FROST and PROFHER trial populations

**DOI:** 10.1186/s13018-023-04319-x

**Published:** 2023-11-08

**Authors:** J. Simpson, A. Keding, S. Spencer, S. Brealey, A. Rangan

**Affiliations:** 1 York Trials Unit, Department of Health Sciences, University of York, York, UK; 2 Hull York Medical School, University of York, York, UK; 3 Health Research Institute, Edge Hill University, Ormskirk, UK

**Keywords:** Factor structure, Frozen shoulder, Proximal humeral fracture, Pain, Function

## Abstract

**Aims:**

Frozen shoulder and proximal humeral fracture can cause pain, stiffness and loss of function. The impact of these symptoms on patients can be measured using the comprehensively validated, 12-item Oxford Shoulder Score (OSS). Evidence suggests that pain and function may have a differential impact on patients’ experience of shoulder conditions, and this may be important for clinical management. We therefore explored the factor structure of the OSS within the UK FROST and PROFHER trial populations.

**Methods:**

We performed exploratory factor analysis (EFA), followed by confirmatory factor analysis (CFA), on baseline UK FROST data from 490 of the 503 trial participants. Data at 6 months post-randomisation were used for 228 of the 250 participants for the PROFHER trial.

**Results:**

UK FROST factor extraction results, using Velicer's Minimum Average Partial and Horn's Parallel Analysis tests, suggested a unifactorial solution, but two factors were weakly indicated by the less reliable ‘Kaiser’s eigenvalue > 1’ and scree tests. We explored this further using EFA. Eight items (2 to 7, 9 and 10) loaded onto a ‘Function’ factor, three on a ‘Pain’ factor (1, 8 and 12) and item 11 cross-loaded. However, one- and two-factor models were rejected in CFA. Factor extraction of PROFHER data at 6 months demonstrated a single first-order factor solution, which was also subsequently rejected in CFA.

**Conclusion:**

Insufficient evidence was found, within the constraints of the data available, to support the use of ‘Pain’ and ‘Function’ sub-scales of the OSS in either patient population.

**Supplementary Information:**

The online version contains supplementary material available at 10.1186/s13018-023-04319-x.

## Introduction

Primary frozen shoulder (adhesive capsulitis) is a common, idiopathic condition of insidious and spontaneous onset, involving progressively escalating pain, glenohumeral joint stiffness and global functional impairment [1]. It is prevalent in middle age, more common in women and in patients with diabetes mellitus [1, 2]. Symptom resolution and functional recovery can take up to three years and may lead to persistent disability [3, 4].

Proximal humeral fractures are common in adults, particularly the elderly and patients with osteoporotic bone [5]. They can be managed conservatively with immobilisation, or surgically with internal fixation or arthroplasty [6]. Shoulder pain and stiffness may persist beyond fracture union or with non-union.

The International Classification of Functioning, Disability and Health (ICF) description of functional health status includes impairments, participation restrictions and activity limitations [7]. Health-related patient-reported outcome measures (PROMs) that assess these limitations are important for evaluating the effectiveness of interventions for shoulder problems.

The Oxford Shoulder Score (OSS) is a widely used PROM for assessing outcomes of treatments for shoulder conditions and was originally conceptualised to be used as a single summary scale [8]. The 12-item single dimension measure has been validated in a range of patient populations [9–11]. Using baseline data from a UK shoulder surgery trial (UKUFF), Dawson and colleagues identified two potentially distinct component scales (pain and function) that could be used as outcomes in practice and research [12]. However, reproducibility in other populations is yet to be established.

The OSS was a patient-centred outcome in two large, UK randomised controlled trials (RCTs): UK FROST compared three interventions for adults with a primary frozen shoulder referred to secondary care [13] and PROFHER compared surgical and non-surgical management of adults with displaced proximal humeral fractures [6]. Our aim was to use these data to examine the dimensional structure of the OSS, to replicate the Dawson and colleagues study, as to whether there are two components of pain and function [12].

## Methods

### Secondary data analysis—UK FROST

The trial included 503 adults with a frozen shoulder recruited from 35 UK hospitals between April 2015 and December 2017. Eligibility criteria are listed in Additional file 1: Table S1 [13]. Patients were randomly assigned to either Early Structured Physiotherapy, manipulation under anaesthesia or arthroscopic capsular release. All participants provided written informed consent. Ethics approval was obtained from the National Research Ethics Service (NRES Committee North East, 14/NE/1176). Full trial details are reported elsewhere [13]. We used baseline data in the analyses.

### Secondary data analysis—PROFHER

The trial included 250 adults with a proximal humeral fracture recruited from 32 UK hospitals between September 2008 and April 2011. Eligibility criteria are listed in Additional file 1: Table S1 [6]. Patients were randomly assigned to either non-operative management (sling immobilisation) or surgery (fracture fixation or humeral head replacement). Ethical approval was obtained from the York Research Ethics Committee (reference number 08/H131/12). Full details of the trial are published elsewhere [6]. Completion of the OSS is based on a recall period of 4 weeks, but as fractures are acute events, it was not possible to collect baseline measurements. We therefore used OSS data collected at 6 months post-randomisation.

### Outcome measures

The OSS is a condition-specific measure focusing on pain and functional limitation of the shoulder following shoulder surgery. It comprises 12-items about symptoms over the past four weeks (items 1, 8, 11 and 12 on pain, the remainder on functionality), each with five response options. Responses are summed to create an overall score ranging from 0 (most severe) to 48 (least severe) [14].

### Analysis

We used factor analysis (FA) to identify potential latent variables, the possible underlying traits that the OSS is measuring [15]. Exploratory Factor Analysis (EFA) was used to identify factors [16] and Confirmatory Factor Analysis (CFA) was used to establish ‘goodness-of-fit’ of the a priori factor structure models, i.e. whether the models explain the data structure well, generated from the EFA results [16].

Data management, descriptive statistics and EFA were conducted using Stata version 16.0 (StataCorp LLC, 4905 Lakeway Drive, College Station, Texas, USA), IBM SPSS Statistics version 26 (UK Head Office, IBM United Kingdom Limited, North Harbour, Portsmouth, Hampshire, UK) and FACTOR version 9.2 [17, 18] software. CFA was performed using LISREL 10.2 (Student) structural equation modelling software (Copyright by Scientific Software International Inc., 1981–2019, Skokie, IL, USA). Analyses were based on polychoric correlation matrices, instead of Pearson correlation coefficients, because of the lack of normality in the ordinal questionnaire data [19, 20].

### EFA

Suitability for factor analysis was tested using Kaiser–Meyer–Olkin (KMO) measure of sampling adequacy (> 0.8) and Bartlett’s test of sphericity (*P* value for *x*^2^ of < 0.01) [21] Principal axis factoring was selected for the extraction method to identify all factors and due to the violation of multivariate normality [22]. A number of common indices were used to determine the appropriate number of factors from the data, which are generally based on the assessment of common variance between items: Velicer’s minimum average partial (MAP) test [23], Horn’s parallel analysis (PA) [24], parallel analysis based on minimum rank factor analysis (PA-MRFA) [25, 26], Hull method [27], scree test [28], and Kaiser’s eigenvalue > 1 [29]. The ‘Common part Accounted For’ measure of fit was used for the Hull method, as this can accompany any factor extraction technique.

We extracted factors using oblique (Promax) rotation to allow correlation between factors, as they cannot be expected to be independent of one another [30]. The association of each OSS item with any identified factor was expressed as pattern matrix loadings. Items with a loading > 0.3 [16] and at least 0.2 greater loading than on other factors to discriminate from them were considered loaded onto a given factor [31]. Internal consistency of the unidimensional and multidimensional OSS, i.e. the degree to which all items measure a common construct, was tested using Cronbach’s alpha [32]; with ≥ 0.7 considered satisfactory internal consistency and > 0.8 reflecting optimal consistency [33].

### CFA

Hypothetical factor models developed using EFA were tested for fit using CFA.

#### UK FROST Models

- *Model 1* tested the single factor solution for all 12 OSS items. Model fit was confirmed by Velicer’s MAP, Horn’s PA, PA-MRFA and Hull method tests. Cronbach’s α: 0.89.
- *Model 2* comprised two first-order factors based on the less reliable Kaiser’s eigenvalue > 1 and scree tests. Items 1, 8 and 12 loaded on the ‘Pain’ factor and the remaining items (including cross-loaded item 11) loaded ‘Function’. Cronbach’s α: 0.66 for ‘Pain’ and 0.90 for ‘Function’.
- *Model 3* replicated Model 2, but with the cross-loaded item 11 loaded on ‘Pain’. Cronbach’s α: 0.71 for ‘Pain’ and 0.88 for ‘Function’.

#### PROFHER Model

- *Model 1* tested the single factor solution for the 12 OSS items, which was supported by results from all factor extraction methods. Cronbach’s α: 0.95.

Due to lack of data normality, we used the diagonally weighted least squares (DWLS) extraction, based on polychoric correlations and asymptomatic covariances [20]. A chi-square test for model fit was conducted and standardised chi-square values reported for each model, with higher values indicating poorer fit. The following goodness-of-fit indices were considered satisfactory: comparative fit index (CFI) > 0.95, goodness-of-fit index (GFI) > 0.95, standardised root mean square residual (SRMSR) < 0.08 and root mean square error of approximation (RMSEA) < 0.1 [34].

## Results

### UK FROST

A complete (orthogonal) baseline dataset was available for 490 (97.4%) of the 503 trial participants. Mean age was 54.3 years (SD 8.2) and 36.7% were male (*n *= 180). Mean OSS total score at baseline was 19.8 (SD 8.2).

#### EFA

KMO (0.92) and Bartlett’s tests (*p* < 0.001) confirmed data suitability for factor analysis. A one-factor solution was supported by the four more reliable extraction methods, with a two-factor model solution suggested by the remaining less reliable tests (Table 1; Additional file 1: Fig. S1 for the scree plot).

**Table 1:** UK FROST factor extraction outcomes

Method	Factors
Velicer’s MAP	1
Horn’s PA	1
PA-MRFA	1
Hull method	1
Scree test	1–2*
Kaiser’s eigenvalue > 1	2

*Borderline 1 or 2 factor scree plot

*MAP* Minimum average partial, *PA* Parallel analysis, *MRFA* Based on minimum rank factor analysis

Using Kaiser’s eigenvalue > 1 rule, eight OSS items (2 to 7, 9 and 10) loaded onto a ‘Function’ factor (eigenvalue 6.39) and three (1, 8 and 12) onto a ‘Pain’ factor (eigenvalue 1.19) (Table 2). Item 11 cross-loaded on both factors.

**Table 2:** UK FROST EFA solution—pattern matrix

OSS item	Factor	
	1 Function	2 Pain
4—Ability to use a knife and fork	.898	− .178
6—Ability to carry a tray with a plate of food	.897	− .147
5—Ability to do household shopping	.862	− .031
3—Ability to go get in and out of a car/public transport	.662	.049
10—Ability to wash and dry under both arms	.650	.133
7—Ability to brush/comb hair with affected arm	.648	.137
9—Ability to hang up clothes with affected arm	.621	.161
2—Ability to get dressed	.559	.145
11—Pain interference with usual work	**.531**	**.342**
1—Pain severity (worst)	− .084	.769
12—Pain interference at night	− .083	.743
8—Pain severity (average)	.205	.537

Rotation converged in 3 iterations

Cross-loaded item in bold text

The ‘Pain’ and ‘Function’ factors were strongly correlated (*r* = 0.67), accounting for 63.2% of the total variance, with the majority (53.2%) attributed to ‘Function’. The single factor, unidimensional solution accounted for 53.2% of the total variance. The one-factor solution with all 12 items had excellent internal consistency (Cronbach’s *α *= 0.89), as did the 8-item (Cronbach’s *α *= 0.88) and 9-item (including item 11) (Cronbach’s *α *= 0.90) ‘Function’ factors. However, internal consistency for the 3-item ‘Pain’ factor was unsatisfactory (Cronbach’s *α *= 0.66) and the 4-item version (including item 11) was only borderline acceptable (Cronbach’s *α *= 0.71).

#### CFA

CFA models were hypothesised from the preceding EFA results. Model 1 is the one-factor unidimensional solution. Models 2 and 3 are two-factor multidimensional solutions, with cross-loaded item 11 incorporated in either ‘Pain’ or ‘Function’. All three CFA models failed goodness-of-fit tests with large normed chi-square values and were rejected based on CFI and RMSEA (Table 3).

**Table 3:** UK FROST CFA results

UK FROST OSS factor model	*X*^2^	d*f*	Normed *X*^2^	CFI	GFI	SRMSR	RMSEA	RMSEA 90% CIs
Model 1 (1 factor) All 12 items	272.83	54	5.05	**0.935**	0.986	0.0683	**0.137**	(0.127; 0.148)
Model 2 (2 factors) Pain = items 1,8,12 Function = all other items	214.40	53	4.05	0.952	0.990	0.0540	**0.121**	(0.111; 0.132)
Model 3 (2 factors) Pain = items 1,8,12,11 Function = all other items	233.33	53	4.40	**0.946**	0.989	0.0581	**0.129**	(0.118; 0.140)

Unsatisfactory results based on standard cut-offs in bold text

*CFI* Comparative fit index, *GFI* Goodness-of-fit index, *SRMSR* Standardised root mean square residual, *RMSEA* Root mean square error of approximation

### PROFHER

An orthogonal dataset of complete OSS scores at the 6-month follow-up was available for 228 (91.2%) of the 250 patients recruited to the study. Of these 228 participants, mean age was 65.7 years old (SD 11.5) and 76.8% were male (*n *= 175). Mean OSS total score at 6 months was 34.5 (SD 10.6).

#### EFA

KMO (0.95) and Bartlett’s tests (*P* value for *x*^2^ of < 0.001) confirmed data suitability for factor analysis. A one-factor solution was confirmed by all extraction methods (Table 4; Additional file 1: Fig. S1 for the scree plot). The single latent factor (eigenvalue 9.15) explained 76.2% of the variance in the data and the unidimensional solution had excellent internal consistency (Cronbach’s *α *= 0.95).

**Table 4:** PROFHER factor extraction outcomes

Method	Factors
Velicer’s MAP	1
Horn’s PA	1
PA-MRFA	1
Hull method	1
Scree test	1
Kaiser’s eigenvalue > 1	1

*MAP* Minimum average partial, *PA* Parallel analysis, *MRFA* Based on minimum rank factor analysis

#### CFA

Similarly to UK FROST analyses, the single factor model for PROFHER was also rejected in CFA, with an unsatisfactory RMSEA ‘goodness-of-fit’ test result (Table 5). The remainder of fit test results were within acceptable limits.

**Table 5:** PROFHER CFA results

PROFHER OSS Factor Model	*X*^2^	d*f*	Normed *X*^2^	CFI	GFI	SRMSR	RMSEA	RMSEA 90% CIs
Model 1 (1 factor) All 12 items	119.08	54	2.21	0.979	0.997	0.0393	**0.154**	(0.139; 0.170)

Unsatisfactory result based on standard cut-off in bold text

*df* Degrees of freedom, *CFI* Comparative fit index, *GFI* Goodness-of-fit index, *SRMSR* Standardised root mean square residual, *RMSEA* Root mean square error of approximation

## Discussion

Multidimensional PROMs comprise components with a differential impact on quality of life and may help understand complex effects of interventions and personalised clinical management. We therefore explored the OSS factor structure for two common shoulder conditions. Results using UK FROST data suggested a two-factor, multidimensional solution, but our hypothetical factor structure models could not confirm this. Analyses using PROFHER data indicated a unidimensional model, but also failed fit testing. There was, therefore, no evidence within the constraints of the data available to support the use of OSS component scales in adults with either primary frozen shoulder or proximal humeral fracture.

Dawson et al. [12] identified pain and function components in the OSS using data from a UK trial of surgical repair of degenerative rotator cuff tears. However, these findings may not be generalisable to the wider population of patients undergoing shoulder surgery that we examined in our study. Dawson et al. [12] and our UK FROST analyses show a consistent pattern of results. Two-factor models tested in both the UKUFF and UK FROST analyses were based on the less reliable scree and eigenvalue extraction methods, with the UKUFF 2-factor solution also supported by Horn’s PA [35]. In each study, similar items loaded on the pain and function scales, with item 11 cross-loaded on both factors. However, our two-factor solution did not strongly discriminate between the closely linked constructs of pain and function. The differential impact of post-surgical pain and function may be more salient for patients with rotator cuff problems compared to those with other conditions, such as frozen shoulder or fractures.

The main difference between the previous study and our analyses is the lack of model fit. Although polychoric matrices were used to address the ordinal OSS data, the two extracted factors were highly correlated and included a cross-loaded item, constituting a weak structure. In addition, the ‘Pain’ factor only included four items and accounted for less than 10% of the overall variance. Overall, factor loadings in our study were slightly weaker, and the sample size 24% smaller, than in the UKUFF analyses. While a strong factor structure may be resistant to sample size variation, the weak two-factor solution from UK FROST may require a larger sample size to achieve model fit [36]. Of note, the proximal humeral fracture cohort contained substantially fewer patients than in the UKUFF analysis which may have influenced model fit. The limited sample size of these secondary data precluded separate sampling for the exploratory and confirmatory analyses, thus some overestimation of fit statistics resulting from using a single source cannot be ruled out.

Participants recruited to UKUFF had symptomatic, degenerative, full-thickness rotator cuff tears, considered suitable for surgery. Eligibility for elective cuff repair (and post-operative immobilisation) means that the UKUFF population may have not included patients with stiffness, as they are generally not listed for surgery but instead receive physiotherapy at that stage. Thus, the UKUFF cohort may have had relatively good pre-operative shoulder movement and functionality at baseline, as evidenced with a mean OSS of 25 compared to 20 in UK FROST. In contrast, UK FROST recruited patients with significantly limited passive external rotation and therefore, by definition, stiff shoulders. Responses to ‘Function’ questions in UK FROST may therefore have been less variable compared to UKUFF. This more homogenous sample may have obscured the distinction between pain and function constructs in the frozen shoulder population.

This paper presents a thorough and rigorous analysis of the baseline UK FROST data using robust methodology. UK FROST recruited over five hundred patients from 35 hospitals in the UK. We are confident that, while it is a marginally smaller sample compared with UKUFF, it should be a representative sample of adults with a frozen shoulder and a sufficiently large dataset with which to perform the analyses.

The secondary analyses from the PROFHER trial have potential limitations. Use of non-baseline study data may interfere with the validity of the factor structures. Data at 6 months was the earliest assessment available because the recall of questions is over the preceding four weeks, which includes pre-fracture status at baseline. This limitation is evidenced with patients at 6 months post-randomisation having relatively ‘healthy’ index shoulders (mean OSS of 34.5), due to the length of time since being treated. The unifactorial solution demonstrated in the PROFHER factor extraction fits with these observations. Further analyses based on item-response theory (Rasch analysis [37]) and using larger data sets may further evaluate the unidimensionality of the OSS.

## Conclusion

We were unable to replicate previous evidence of pain and function component scales in the OSS in either patient population. Although loading patterns were similar using data from frozen shoulder patients (UK FROST) and for those with a rotator cuff tear (UKUFF), our models could not confirm this. There is therefore insufficient evidence, within the constraints of the data available, to recommend the use of OSS component scales in frozen shoulder or proximal humeral fracture populations. Instead, within these settings, we would recommend using the PROM as a single summary scale as it was originally conceptualised.

### Supplementary Information

**Additional file 1: Table S1**. Eligibility criteria for UK FROST. **Table S2.** Eligibility criteria for PROFHER. **Fig. S1.** Factor extraction scree plot for baseline UK FROST data. **Fig. S2**. Factor extraction scree plot for PROFHER data at 6 months.

## Data Availability

Supporting data is available.
