# Supplementary material for: Factor structure of the Oxford Shoulder Score: secondary analyses of the UK FROST and PROFHER trial populations
Source: J Orthop Surg Res. 2023 Nov 8;18:846. doi: 10.1186/s13018-023-04319-x (PMC10631035; doi:10.1186/s13018-023-04319-x)
Supplement: Supplementary file 1 — Additional file 1: Table S1. Eligibility criteria for UK FROST. Table S2. Eligibility criteria for PROFHER. Fig. S1. Factor extraction scree plot for baseline UK FROST data. Fig. S2. Factor extraction scree plot for PROFHER data at 6 months. [file 13018_2023_4319_MOESM1_ESM.docx]

**Supplementary Material**

**Table S1** Eligibility criteria for UK FROST

| **UK FROST** | |
| --- | --- |
| **Inclusion Criteria** | **Exclusion Criteria** |
| Aged 18 years or older | Bilateral concurrent frozen shoulder |
| Unilateral frozen shoulder | Secondary causes |
| Restricted passive external rotation in the affected shoulder to <50% of the other shoulder | Any contraindication to a trial treatment, including being unfit for anaesthesia or corticosteroid injection |
| Radiographs excluding other pathology | Insufficient mental capacity to understand the trial or instructions |
|  | Not resident within trial site catchment area |

**Table S2** Eligibility criteria for PROFHER

| **PROFHER** | |
| --- | --- |
| **Inclusion Criteria** | **Exclusion Criteria** |
| Aged 16 years or older | Dislocation of the injured shoulder |
| Presenting within 3 weeks of sustaining a displaced fracture of the proximal humerus, involving the surgical neck | Open fracture |
|  | Clear indication for surgery e.g. severe soft-tissue compromise |
| Displacement sufficient to consider surgery | Multiple upper limb fractures |
|  | Pathological fracture (other than osteoporotic) |
|  | Terminal illness or comorbidities precluding anaesthesia or surgery |
|  | Insufficient mental capacity to understand the trial or instructions |
|  | Not resident within hospital catchment area |

**Figure S1** Factor extraction scree plot for baseline UK FROST data.

**Figure S2** Factor extraction scree plot for PROFHER data at 6 months.
